# Supplementary material for: COZOID: contact zone identifier for visual analysis of protein-protein interactions
Source: BMC Bioinformatics. 2018 Apr 6;19:125. doi: 10.1186/s12859-018-2113-6 (PMC5889581; doi:10.1186/s12859-018-2113-6)
Supplement: Supplementary file 2 — Software build. Executable binary file of the software tool. (ZIP 118,784 kb) [file 12859_2018_2113_MOESM2_ESM.zip › cozoid/java/docs/beansbinding-1.2.1-doc.zip!/org/jdesktop/swingbinding/JComboBoxBinding.html]

JComboBoxBinding (Beans Binding)


---


|  |  |  |
| --- | --- | --- |
| **Overview** \| **Package** \| **Class** \| **Use** \| **Tree** \| **Deprecated** \| **Index** \| **Help** | |  |
| PREV CLASS   **NEXT CLASS** | **FRAMES**    **NO FRAMES**     **All Classes** |  |
| SUMMARY: NESTED \| FIELD \| CONSTR \| METHOD | DETAIL: FIELD \| CONSTR \| METHOD |  |


---


## org.jdesktop.swingbinding Class JComboBoxBinding<E,SS,TS>

```
java.lang.Object
  org.jdesktop.beansbinding.Binding<SS,SV,TS,TV>
      org.jdesktop.beansbinding.AutoBinding<SS,java.util.List<E>,TS,java.util.List>
          org.jdesktop.swingbinding.JComboBoxBinding<E,SS,TS>
```

**Type Parameters:**: `E` - the type of elements in the source `List`: `SS` - the type of source object (on which the source property resolves to `List`): `TS` - the type of target object (on which the target property resolves to `JComboBox`)

---

``` public final class JComboBoxBinding<E,SS,TS> extends AutoBinding<SS,java.util.List<E>,TS,java.util.List> ```

Binds a `List` of objects to act as the items of a `JComboBox`.
Each object in the source `List` is an item in the `JComboBox`.
Instances of `JComboBoxBinding` are obtained by calling one of the
`createJComboBoxBinding` methods in the `SwingBindings` class.

Here is an example of creating a binding from a `List` of `Country`
objects to a `JComboBox`:

```
    // create the country list
    List countries = createCountryList();

    // create the binding from List to JComboBox
    JComboBoxBinding cb = SwingBindings.createJComboBoxBinding(READ, countries, jComboBox);

    // realize the binding
    cb.bind();
```

If the `List` is an instance of `ObservableList`, then changes to
the `List` contents (such as adding, removing or replacing an object)
are reflected in the `JComboBox`. **Important:** Changing the contents
of a non-observable `List` while it is participating in a
`JComboBoxBinding` is unsupported, resulting in undefined behavior and
possible exceptions.

`JComboBoxBinding` requires
extra clarification on the operation of the
`refresh` and `save` methods and the meaning of the update
strategy. The target property of a `JComboBoxBinding` is not the
target `JComboBox` property provided in the constructor, but rather a
private synthetic property representing the `List` of objects to show
in the target `JComboBox`. This synthetic property is readable/writeable
only when the `JComboBoxBinding` is bound and the target `JComboBox`
property is readable with a `non-null` value.

It is this private synthetic property on which the `refresh` and
`save` methods operate; meaning that these methods simply cause syncing
between the value of the source `List` property and the value of the
synthetic target property (representing the `List` to be shown in the
target `JComboBox`). These methods do not, therefore, have anything to do
with refreshing *values* in the `JComboBox`. Likewise, the update
strategy, which simply controls when `refresh` and `save` are
automatically called, also has nothing to do with refreshing *values*
in the `JComboBox`.

**Note:** At the current time, the `READ_WRITE` update strategy
is not useful for `JComboBoxBinding`. To prevent unwanted confusion,
`READ_WRITE` is translated to `READ` by `JComboBoxBinding's`
constructor.

`JComboBoxBinding` works by installing a custom model on the target
`JComboBox`, as appropriate, to represent the source `List`. The
model is installed on a target `JComboBox` with the first succesful call
to `refresh` with that `JComboBox` as the target. Subsequent calls
to `refresh` update the elements in this already-installed model.
The model is uninstalled from a target `JComboBox` when either the
`JComboBoxBinding` is unbound or when the target `JComboBox` property
changes to no longer represent that `JComboBox`. Note: When the model is
uninstalled from a `JComboBox`, it is replaced with a `DefaultComboBoxModel`,
in order to leave the `JComboBox` functional.

Some of the above is easier to understand with an example. Let's consider
a `JComboBoxBinding` (`binding`), with update strategy
`READ`, between a property representing a `List` (`listP`)
and a property representing a `JComboBox` (`jComboBoxP`). `listP`
and `jComboBoxP` both start off readable, referring to a `non-null`
`List` and `non-null` `JComboBox` respectively. Let's look at
what happens for each of a sequence of events:

| Sequence | Event | Result |
| --- | --- | --- |
| 1 | explicit call to `binding.bind()` | - synthetic target property becomes readable/writeable   - `refresh()` is called   - model is installed on target `JComboBox`, representing list of objects |
| 2 | `listP` changes to a new `List` | - `refresh()` is called   - model is updated with new list of objects |
| 3 | `jComboBoxP` changes to a new `JComboBox` | - model is uninstalled from old `JComboBox` |
| 4 | explicit call to `binding.refresh()` | - model is installed on target `JComboBox`, representing list of objects |
| 5 | `listP` changes to a new `List` | - `refresh()` is called   - model is updated with new list of objects |
| 6 | explicit call to `binding.unbind()` | - model is uninstalled from target `JComboBox` |

Notice that in step 3, when the value
of the `JComboBox` property changed, the new `JComboBox` did not
automatically get the model with the elements applied to it. A change to the
target value should not cause an `AutoBinding` to sync the target from
the source. Step 4 forces a sync by explicitly calling `refresh`.
Alternatively, it could be caused by any other action that results
in a `refresh` (for example, the source property changing value, or an
explicit call to `unbind` followed by `bind`).

In addition to binding the items of a `JComboBox`, it is possible to
bind to the selected item of a `JComboBox`.
See the list of 
interesting swing properties in the package summary for more details.

---

**Nested Class Summary**

**Nested classes/interfaces inherited from class org.jdesktop.beansbinding.AutoBinding**

`AutoBinding.UpdateStrategy`

**Nested classes/interfaces inherited from class org.jdesktop.beansbinding.Binding**

`Binding.SyncFailure, Binding.SyncFailureType, Binding.ValueResult<V>`


| **Constructor Summary** | |
| --- | --- |
| `protected` | `JComboBoxBinding(AutoBinding.UpdateStrategy strategy, SS sourceObject, Property<SS,java.util.List<E>> sourceListProperty, TS targetObject, Property<TS,? extends javax.swing.JComboBox> targetJComboBoxProperty, java.lang.String name)`             Constructs an instance of `JComboBoxBinding`. |


| **Method Summary** | |
| --- | --- |
| `protected  void` | `bindImpl()`             Called by `Binding.bind()` to allow subclasses to initiate binding. |
| `protected  void` | `unbindImpl()`             Called by `Binding.unbind()` to allow subclasses to uninitiate binding. |

**Methods inherited from class org.jdesktop.beansbinding.AutoBinding**

`getUpdateStrategy, paramString, sourceChangedImpl, targetChangedImpl`

**Methods inherited from class org.jdesktop.beansbinding.Binding**

`addBindingListener, addPropertyChangeListener, addPropertyChangeListener, bind, bindUnmanaged, firePropertyChange, getBindingListeners, getConverter, getName, getPropertyChangeListeners, getPropertyChangeListeners, getSourceNullValue, getSourceObject, getSourceProperty, getSourceUnreadableValue, getSourceValueForTarget, getTargetNullValue, getTargetObject, getTargetProperty, getTargetValueForSource, getValidator, isBound, isManaged, isSourceUnreadableValueSet, notifySynced, notifySyncFailed, refresh, refreshAndNotify, refreshAndNotifyUnmanaged, refreshUnmanaged, removeBindingListener, removePropertyChangeListener, removePropertyChangeListener, save, saveAndNotify, saveAndNotifyUnmanaged, saveUnmanaged, setConverter, setManaged, setSourceNullValue, setSourceObject, setSourceObjectUnmanaged, setSourceProperty, setSourceUnreadableValue, setTargetNullValue, setTargetObject, setTargetObjectUnmanaged, setTargetProperty, setValidator, throwIfBound, throwIfManaged, throwIfUnbound, toString, unbind, unbindUnmanaged, unsetSourceUnreadableValue`

**Methods inherited from class java.lang.Object**

`clone, equals, finalize, getClass, hashCode, notify, notifyAll, wait, wait, wait`

**Constructor Detail**

### JComboBoxBinding

```
protected JComboBoxBinding(AutoBinding.UpdateStrategy strategy,
                           SS sourceObject,
                           Property<SS,java.util.List<E>> sourceListProperty,
                           TS targetObject,
                           Property<TS,? extends javax.swing.JComboBox> targetJComboBoxProperty,
                           java.lang.String name)
```

:   Constructs an instance of `JComboBoxBinding`.

    **Parameters:**: `strategy` - the update strategy: `sourceObject` - the source object: `sourceListProperty` - a property on the source object that resolves to the `List` of elements: `targetObject` - the target object: `targetJComboBoxProperty` - a property on the target object that resolves to a `JComboBox`: `name` - a name for the `JComboBoxBinding` **Throws:**: `java.lang.IllegalArgumentException` - if the source property or target property is `null`


**Method Detail**

### bindImpl

```
protected void bindImpl()
```

:   **Description copied from class: `Binding`**
:   Called by `Binding.bind()` to allow subclasses to initiate binding.
    Subclasses typically need not install `PropertyStateListeners`
    on the source property and target property as they will be notified
    by calls to `Binding.sourceChangedImpl(org.jdesktop.beansbinding.PropertyStateEvent)` and `Binding.targetChangedImpl(org.jdesktop.beansbinding.PropertyStateEvent)`
    when the source and target properties change respectively.

    :   **Overrides:**: `bindImpl` in class `AutoBinding<SS,java.util.List<E>,TS,java.util.List>`
    :   **See Also:**: `Binding.unbindImpl()`

---


### unbindImpl

```
protected void unbindImpl()
```

:   **Description copied from class: `Binding`**
:   Called by `Binding.unbind()` to allow subclasses to uninitiate binding.

    :   **Overrides:**: `unbindImpl` in class `AutoBinding<SS,java.util.List<E>,TS,java.util.List>`
    :   **See Also:**: `Binding.bindImpl()`


---


|  |  |  |
| --- | --- | --- |
| **Overview** \| **Package** \| **Class** \| **Use** \| **Tree** \| **Deprecated** \| **Index** \| **Help** | |  |
| PREV CLASS   **NEXT CLASS** | **FRAMES**    **NO FRAMES**     **All Classes** |  |
| SUMMARY: NESTED \| FIELD \| CONSTR \| METHOD | DETAIL: FIELD \| CONSTR \| METHOD |  |


---
